# Beliefs about Breast Cancer among Women in the Western Amazon: A Population-Based Study

**DOI:** 10.31557/APJCP.2019.20.2.469

**Published:** 2019

**Authors:** Marla Presa Raulino Schilling, Ilce Ferreira da Silva, Simone Perufo Opitz, Maria Fernanda de Sousa Oliveira Borges, Rosalina Jorge Koifman, Sergio Koifman

**Affiliations:** 1 *National School of Public Health, Oswaldo Cruz Foundation, Postgraduate Program in Public Health and Environment, *; 2 *Federal University of Acre, Postgraduate Program in Public Health, Brazil. *

**Keywords:** Breast cancer, risk factors, epidemiology, beliefs

## Abstract

**Objective:**

Evaluate the beliefs about the risk factors for breast cancer in a population of women from the western Amazon and determine the factors associated with the higher belief scores presented by this population.

**Methods:**

A population-based cross-sectional study included 478 women aged **>**40 years residing in Rio Branco, Acre, Brazil. An American Cancer Society questionnaire was applied to assess the knowledge, attitudes, and beliefs about breast cancer.

**Results:**

The main beliefs about the risk factors for breast cancer were breast trauma (95%), use of underwire bra (58.5%), and a high number of sexual partners (55.5%). Women from younger age groups presented higher belief scores (Bcoefficient: –0.04, 95% CI: –0.07; –0.01) than those of women from older age groups. A strong association was noted between high knowledge scores of risk factors and signs/symptoms of the disease and high belief scores in the study group (Bcoefficient:0.33;95%CI:0.28;0.38).

**Conclusion:**

The results indicate the existence of important beliefs related to the risk factors for breast cancer. Women from younger age groups, women who have seen a gynecologist in the past 2 years, and women who had more knowledge about the risk factors and signs and symptoms of breast cancer had higher belief scores.

## Introduction

Breast cancer is a severe public health problem in Brazil and worldwide. Breast cancer is the most common cancer among women (second only to non-melanoma skin cancer) and has the highest mortality rates (Ferlay et al., 2015). This cancer is stigmatized because it involves a multisymbolic female organ, may be fatal, and treatment (mastectomy) typically involves a body-mutilating procedure. Therefore, breast cancer is surrounded by strong myths, fears, and connotations that are beyond the clinical understanding of the disease (Remennick, 2006).

Early detection of breast cancer by regular mammographic screening is one of the most effective methods to promote early treatment and increase the survival rate. However, the responsibility of women for achieving and maintaining good health conditions, the importance they attribute to healthy behaviors and preventive actions, the way they experience the disease, their expectations about the resolution of the problem, and their past experiences can significantly affect their health behaviors (Fugita and Gualda, 2006).

Kleinman (1981) warns of the need to consider health care in different societies as socially organized responses that are part of a cultural system, which he refers to as the “health care system”. The health care system and other cultural systems include a set of beliefs about the causes of illnesses; rules governing treatment choices and evaluations; and socially legitimized statuses, roles, power relations, interaction models, and institutions.

Studies conducted with populations from different cultures indicate the existence of a set of beliefs about the causes of breast cancer (Jones et al., 2011; Lizama et al., 2016; Morse et al., 2014; Opoku et al., 2012; Sobani et al., 2012; Wang et al., 2010). In Australia, a population-based study of 3,005 women found that 54.8% believed that the use of deodorants increased the risk of breast cancer (Jones et al., 2011). In Pakistan, a study demonstrated that 32.7% of women aged 18 to 70 years believed that wearing black clothes increases the risk of breast cancer (Sobani et al., 2012). Other factors still not widely recognized by the scientific community, such as stress (Jones et al., 2011; Lee-Lin et al., 2007; Lizama et al., 2016; Wang et al., 2010) and breast trauma, have been considered by women of different cultures as risk factors for the disease (El-Shinawi et al., 2013; Grunfeld et al., 2002; Jones et al., 2011).

The concept of knowledge and beliefs used in the present study is based on those established by Hume (2000), which defines that knowledge and belief (synonymous of faith, assent, opinion, and “probability”) are independent epistemologically and correspond to distinct cognitive abilities, and the ability to generate beliefs is complementary to the ability to generate knowledge. Leventhal’s theory of self-regulation, which is often referred to as the “common-sense model,” postulates that individuals’ cognitive representations of a disease guide health behaviors, including adherence to treatment and changes in health practices (Leventhal et al., 1980; Leventhal et al., 1997). Therefore, beliefs play a significant role in determining how women understand and explain breast cancer, and this understanding may have a strong impact on behaviors related to breast cancer screening(Kwok and Sullivan, 2006; Thomson et al., 2014). In addition, sociocultural and psychological barriers that may affect the decision to seek health care include denial of personal risk, fatalism attributed to the disease, lack of confidence in cancer treatment, and fear of becoming a burden to the family (Fugita and Gualda, 2006; Remennick, 2006). In this sense, understanding the beliefs about breast cancer and associated factors from a cultural perspective is essential, especially in culturally mixed populations, such as the Brazilian population. 

The city of Rio Branco, Acre, is located in the western Brazilian Amazon. The first studies evaluating breast cancer in this population were conducted in 2011. The results indicated that this cancer had the highest incidence and mortality rates (Nakashima et al., 2011; Nakashima et al., 2012) among women, and that cancer-specific survival was high (Fujimoto et al., 2017). In Brazil, no quantitative population-based studies to date analyzed the set of beliefs of women about the etiology of breast cancer. Therefore, the objective of this study is to evaluate the set of beliefs about breast cancer risk factor and the associated factors among women aged > 40 years living in Rio Branco, Acre, Brazil.

## Materials and Methods

This study is a subproject of a leading study titled “Brazilian Breast Health Knowledge, Attitudes, and Practices(KAP) Survey,” from the National School of Public Health of the Oswaldo Cruz Foundation. 

A population-based cross-sectional study included a sample population obtained by two-stage cluster sampling as detailed in previous studies (Borges et al., 2012; Schilling et al., 2017). Briefly, from 250 census tracts defined by the Brazilian Institute of Geography and Statistics(IBGE) for the 2000 census (IBGE, www.ibge.gov.br), 35 census tracts that were previously used by the National Household Sample Survey were selected in 2006. Of these data, 25 households within each sector were randomly selected with the final selection of 875 households. Eventual losses and refusals were accounted for by selecting an additional 15% of households, totaling 977 households, which represent the population geographically distributed in the municipality of Rio Branco. From 1,516 residents aged > 18 years in the selected households, all women aged > 40 years (n = 478) were interviewed. Whenever a selected household was closed or the resident was absent, the interviewer would make at least three different attempts (at different days and times) to contact the resident in the household.

Data were collected from July 2012 to March 2013 by trained interviewers. For quality control of data collection, weekly team meetings were conducted to clarify doubts and reinforce the training provided to interviewers. In addition, each interviewer was accompanied by a researcher at different stages of data collection. 

A semi-structured questionnaire developed by the American Cancer Society translated and back-translated into Portuguese was used for data collection. The instrument was organized in modules, which include data on sociodemographic and epidemiological characteristics, health care, women’s experiences and understanding of the disease, knowledge about breast cancer risk factors, signs, and symptoms; and beliefs about risk factors.

Belief variable was assessed using questions related to the set of beliefs on the etiology of breast cancer. The study population was asked whether “hit in the breast”; “having many sexual partners”; “food additives”; “consumption of genetically modified foods”; “stress”; “depression, anger, bitterness, or resentment”; “great life disappointments”; “oral sex” and “using underwire bra” would increase the probability of breast cancer.

Responses regarding the beliefs about the risk factors for breast cancer were scored from 0 to 2. A score of “0” was assigned to the belief that the analyzed factor did not increase the risk of developing breast cancer. A score of “1” was assigned to the belief of a moderate increase in the risk of breast cancer. A score of “2” corresponded to the belief that the analyzed factor strongly increased the risk of developing breast cancer. Women’s beliefs about breast cancer ranged from 0 to 18 points. The score was evaluated continuously and subsequently categorized. 

The belief variable was categorized using tertiles, such that a low belief score was defined by the first tertile (< 7 points), an intermediate belief score was defined by the second tertile(7 to 11 points), and a high belief score was defined by the third tertile(> 11 points). 

The sociodemographic and epidemiological variables studied were place of residence(urban sector/rural sector), age(continuous/stratified into 40–49, 50–59, 60–69, and >70 years), schooling (continuous/stratified into 0–8, 9–12, and > 12 years), marital status(without a partner/with a partner), history of pregnancy (yes/no), breastfeeding over 1 month(yes/no), menopausal status (yes/no), and hormone replacement therapy(yes/no). With regard to health care, Pap smear testing(yes/no), types of health services used (only the Unified Health System (SUS), without SUS, or mixed services), and seeing a gynecologist in the past 2 years (yes/no) were evaluated. The interviewees were asked about their experiences and understanding of breast cancer by using the following questions: “Have any of your immediate or extended family members ever had breast cancer?” (yes/no), “Would you say your chance of getting breast cancer is low, moderate or high?” (low/moderate/high) “If you had breast cancer would you want to know?” (yes/no), “Do you think that a woman who has never had any family members with breast cancer can get breast cancer?” (yes/no), “How likely do you think it is that someone will die if they get breast cancer?”(low probability/probably/fatal disease), “What extent do you think that early detection of breast cancer will influence a person´s chance of surviving the breast cancer?”(a lot/not much/nothing), “Do you think you can have breast cancer without having a lump or nodule?” (yes/no), “If a woman finds a lump or nodule in her breast, would you say the chances of it being breast cancer are low, moderate or high?” (low/moderate/high). Knowledge level about breast cancer was evaluated by scoring the knowledge of the risk factors and disease signs/symptoms, as previously described (Schilling et al., 2017).

In the statistical analysis of the data, the frequency distribution of the questions that composed the outcome variable (belief score) was determined. Then, correlation between independent variables and belief’s score was determined by comparing the means using Student’s t-test for independent samples. Associations between independent variables and belief’s score at a significance level of 5% were analyzed using simple linear regression to assess the effect of the independent variables on the belief score. Subsequently, multiple linear regression was used to evaluate the relationship between the belief score of risk factors for breast cancer and the following factors: sociodemographic and epidemiological characteristics; health care; breast cancer experiences and understanding; and knowledge about risk factors and disease signs/symptoms adjusted for age and schooling. Pearson chi-square test was used to analyze the categorized outcomes (high, intermediate, and low scores).

All statistical analyses were conducted using the SPSS statistical package version 20.0. The Human Research Ethics Committee of the National School of Public Health (CEP-ENSP) approved the present study, and informed consent was obtained from all study participants.

## Results

The sample consisted of 478 women aged **>**40 years (mean age of 55.6±11.07 years), and the most common age group was 40 to 49 years(35.6%). Of the total sample, 93.1% women lived in urban areas, 10.3% had more than 12 years of schooling, and 96.7% had a history of pregnancy. Approximately 75% reported having postmenopausal status, of whom 77.6% did not use hormone replacement therapy. The Brazilian SUS was used by 66.5% of the participants, and 60.3% of the sample reported seeing a gynecologist in the past 2 years. 

Approximately 11.0% of women reported knowing someone with breast cancer. Among the interviewees, 9.5% believed they had a high probability of developing the disease, 92.4% would want to know if they had breast cancer, and 90.5% responded that a woman who did not have relatives with breast cancer could get the disease. Approximately 25% of the respondents thought that the disease was fatal, 51% believed that a woman could have breast cancer without having a lump or nodule in the breast, and 32.1% reported that the likelihood of a breast lump being breast cancer was high.

Almost all respondents (95%) thought that breast trauma increased the risk of developing breast cancer. Most women responded that psychological/emotional factors such as stress (68.9%); depression, anger, or bitterness (62.5%); and personal disappointment (55.8%) might increase the risk of breast cancer. Approximately 40% of the interviewees believed that a high number of sexual partners significantly increased the risk of the disease. Moreover, 58.5% of the study sample thought that the use of underwire bra was a risk factor for breast cancer (Table 1).

The mean belief score was 9.29±4.01 points (median=9 points). Age, seeing a gynecologist in the past 2 years, wanting to know if they had breast cancer, belief that it is possible to get breast cancer without having a lump or nodule in the breast, and the belief that the likelihood of a breast lump being breast cancer is high, were statistically associated with beliefs were. Women with higher belief scores presented statistically higher knowledge scores. All variables maintained significant correlation even after adjusting by age and schooling (Table 2).

**Table 1:** Distribution of Beliefs about Risk Factors for Breast Cancer among Women aged ≥40 years living in Rio Branco, Acre, Brazil, from 2012 to 2013

Beliefs	*Not at all (%)	*A little (%)	*A lot (%)
Being hit in the breast increase the risk of breast cancer?	24 (5)	59 (12.4)	394 (82.6)
Having many sexual partners increase the risk of breast cancer?	212 (44.4)	77 (16.1)	188 (39.4)
Food additives increase the risk of breast cancer?	159 (33.5)	126 (26.5)	190 (40)
Consumption of genetically modified foods increases the risk of breast cancer?	184 (39)	135 (28.6)	153 (32.4)
Stress aumenta o risco de câncer de mama?	149 (31.2)	61 (12.8)	268 (56.1)
Depression, anger, bitterness, or resentment increases the risk of breast cancer?	179 (37.5)	91 (19.1)	207 (43.4)
Great life disappointments increase the risk of breast cancer?	211 (44.2)	89 (18.7)	177 (37.1)
Oral sex increases the risk of breast cancer?	336 (70.7)	58 (12.2)	81 (17.1)
Using underwire bra increase the risk of breast cancer?	198 (41.5)	141 (29.6)	138 (28.9)

*, Total may change due to missing values;**, Adjusted by age and education

**Table 2:** Distribution of Breast Cancer Beliefs and Associated Factors Among Women Aged ≥ 40 Years from Western Amazon, Brazil

Variable	*N (%)		Belief score				βcoefficient (95% CI)			
			Mean (SD)		Pvalue		Crude		**Adjusted	
Sector										
Urban	445 (93.1)		9.30 (4.05)		0.867		0.12 (-1.30;1.54)		0.10 (-1.32;1.53)	
Rural	33 (6.9)		9.18 (3.63)							
Age										
40 a 49	170 (35.6)		9.53 (4.10)		0.005		-0.04 (-0.07;-0.01)		-0.04 (-0.07;-0.01)	
50 a 59	143 (29.9)		9.94 (3.58)							
60 a 69	103 (21.5)		8.81 (4.28)							
≥70	62 (13.0)		7.93 (3.99)							
Educational level (completed years)										
0-8	285 (59.6)		9.24 (4.21)		0.533		0.01 (-0.08; 0.09)		0.02 (-0.53;0.58)	
9-12	144 (30.1)		9.18 (3.79)							
>12	49 (10.3)		9.90 (3.49)							
Marital status										
No partner	240 (50.3)		8.97 (4.09)		0.085		0.64 (-0.08;1.37)		0.50 (-0.23;1.24)	
A partner	237 (49.7)		9.62 (3.93)							
Pregnancy										
Yes	462 (96.7)		9.28 (3.99)		0.595		-0.58 (-2.72;1.56)		-0.49 (-2.62;1.64)	
No	16 (3.3)		9.86 (4.60)							
Breast feeding for more than 1 month										
Yes	416 (90.8)		9.25 (4.03)		0.456		-0.48 (-1.75;0.79)		-0.52 (-1.79;0.75)	
No	42 (9.2)		9.74 (3.68)							
Menopause										
Yes	356 (74.5)		9.36 (4.03)		0.578		0.24 (-0.60;1.07)		1.20 (0.20;2.19)	
No	122 (25.5)		9.12 (3.98)							
Hormonal therapy										
Yes	107 (22.4)		9.54 (3.94)		0.503		0.30 (-0.58;1.18)		0.47 (-0.42;1.36)	
No	370 (77.6)		9.24 (4.04)							
Having undergone a Pap-test										
Yes	434 (90.8)		9.39 (3.97)		0.097		1.07 (-0.19;2.32)		0.73 (-0.56;2.03)	
No	44 (9.2)		8.33 (4.37)							
Health system used										
Public only	317 (66.5)		9.25 (4.11)		0.722		0.14 (-0.63;0.92)		0.25 (-0.59;1.10)	
Private and/or mix	160 (33.5)		9.39 (3.83)							
Have you had a consultation with a gynecologist in the last 2 years?										
Yes	286 (60.3)		9.71 (3.83)		0.003		1.13 (0.38;1.87)		0.99 (0.20;1.78)	
No	188 (39.7)		8.58 (4.15)							
Have any of your immediate or extended family members ever had breast cancer?										
Yes	53 (11.1)		9.54 (3.60)		0.643		0.27 (-0.88;1.43)		0.25 (-0.91;1.40)	
No	425 (88.9)		9.26 (4.07)							
Would you say your chance of getting breast cancer is low, moderate or high?										
Low	298 (65.6)		9.28 (3.95)		0.313		0.27 (-0.29;0.83)		0.19 (-0.38;0.76)	
Moderate	113 (24.9)		9.14 (4.27)							
High	43 (9.5)		10.22 (3.31)							
If you had breast cancer would you want to know?										
Yes	439 (92.4)		9.39 (4.02)		0.036		1.48 (0.09;2.86)		1.49 (0.11;2.86)	
No	36 (7.6)		7.91 (3.80)							
Do you think that a woman who has never had any family members with breast cancer can get breast cancer?									
Yes	427 (90.5)	9.43 (3.98)		0.175		0.86 (-0.38;2.10)		0.74 (-0.51;1.99)	
No	45 (9.5)	8.57 (4.05)							
How likely do you think it is that someone will die if they get breast cancer?									
Fatal disease	114 (24.2)	9.61 (4.04)		0.577		0.26 (-0.22;0.74)		0.30 (-0.18;0.79)	
Probably	200 (42.5)	9.35 (3.93)							
Low probability	157 (33.3)	9.09 (4.06)							
What extent do you think that early detection of breast cancer will influence a person´s chance of surviving the breast cancer?									
A lot	398 (84.0)	9.29 (4.02)		0.781		0.14 (-0.86;1.15)		0.08 (-0.93;1.08)	
Not much/nothing; a little/none	76 (16.0)	9.15 (4.04)							
Do you think you can have breast cancer without having a lump or nodule?									
Yes	232 (50.9)	9.74 (3.85)		0.007		1.03 (0.29;1.78)		1.05 (0.30;1.80)	
No	224 (49.1)	8.70 (4.13)							
If a woman finds a lump or nodule in her breast, would you say the chances of it being breast cancer are low, moderate or high?									
High	151 (32.1)	10.32 (4.04)		<0.001		0.79 (0.22;1.35)		0.79 (0.22;1.35)	
Moderate	259 (55.0)	8.75 (3.93)							
Low	61 (13.0)	9.25 (3.87)							
Knowledge									
High	150 (32.3)	11.48 (3.30)		<0.001		0.34 (0.29;0.38)		0.33 (0.28;0.38)	
Moderate	194 (41.7)	9.85 (3.39)							
Low	121 (26.0)	6.59 (3.74)							

**Table 3:** Distribution of Level of Breast Cancer Beliefs According to Selected Associated Factors among Women Aged ≥ 40 Years from Western Amazon, Brazil

Variable	*N (%)	**Beliefs (degree)				
		*High N (%)		*Intermediate N (%)	*Low N (%)	Pvalue
Sector						
Urban	445 (93.1)	140 (93.3)		182 (93.8)	110 (90.9)	0.602
Rural	33 (6.9)	10 (6.7)		12 (6.2)	11 (9.1)	
Age						
40 a 49	170 (35.6)	59 (39.3)		65 (33.5)	43 (35.5)	0.016
50 a 59	143 (29.9)	49 (32.7)		66 (34.0)	25 (20.7)	
60 a 69	103 (21.5)	32 (21.3)		34 (17.5)	32 (26.4)	
≥70	62 (13.0)	10 (6.7)		29 (14.9)	21 (17.4)	
Educational level (completed years)						
0-8	285 (59.6)	91 (60.7)		111 (57.2)	76 (62.8)	0.517
9-12	144 (30.1)	45 (30.0)		57 (29.4)	36 (29.8)	
>12	49 (10.3)	14 (9.3)		26 (13.4)	09 (7.4)	
Marital status						
No partner	240 (50.3)	74 (49.3)		94 (48.7)	66 (54.5)	0.571
A partner	237 (49.7)	76 (50.7)		99 (51.3)	55 (45.5)	
Pregnancy history						
Yes	462 (96.7)	145 (96.7)		187 (96.4)	119 (98.3)	0.59
No	16 (3.3)	05 (3.3)		07 (3.6)	02 (1.7)	
Breast feeding for more than 1 month						
Yes	416 (90.8)	130 (90.3)		165 (88.7)	110 (94.0)	0.301
No	42 (9.2)	14 (9.7)		21 (11.3)	07 (6.0)	
Menopause						
Yes	356 (74.5)		114 (76.0)	144 (74.2)	88 (72.7)	0.826
No	122 (25.5)		36 (24.0)	50 (25.8)	33 (27.3)	
Hormonal therapy						
Yes	107 (22,4)		32 (21.3)	50 (25.8)	20 (16.7)	0.162
No	370 (77,6)		118 (78.7)	144 (74.2)	100 (83.3)	
Having undergone a Pap-test						
Yes	434 (90.8)		140 (93.3)	179 (92.3)	103 (85.1)	0.043
No	44 (9.2)		10 (6.7)	15 (7.7)	18 (14.9)	
Health system used						
Public only	317 (66.5)		106 (70.7)	118 (61.1)	87 (71.9)	0.073
Private and/or mix	160 (33.5)		44 (29.3)	75 (38.9)	34 (28.1)	
Have you had a consultation with a gynecologist in the last 2 years?						
Yes	286 (60.3)		98 (66.2)	123 (63.7)	59 (49.2)	0.009
No	188 (39.7)		50 (33.8)	70 (36.3)	61 (50.8)	
Have any of your immediate or extended family members ever had breast cancer?						
Yes	53 (11.1)		17 (11.3)	24 (12.4)	11 (9.1)	0.666
No	425 (88.9)		133 (88.7)	170 (87.6)	110 (90.9)	
Would you say your chance of getting breast cancer is low, moderate or high?						
Low	298 (65.6)		89 (62.2)	130 (69.9)	70 (62.5)	0.172
Moderate	113 (24.9)		39 (27.3)	37 (19.9)	35 (31.2)	
High	43 (9.5)		15 (10.5)	19 (10.2)	07 (6.2)	
If you had breast cancer would you want to know?						
Yes	439 (92.4)		143 (96.0)	176 (91.7)	108 (89.3)	0.102
No	36 (7.6)		06 (4.0)	16 (8.3)	13 (10.7)	
Do you think that a woman who has never had any family members with breast cancer can get breast cancer?						
Yes	427 (90.5)		140 (93.3)	175 (91.6)	100 (84.7)	0.046
No	45 (9.5)		10 (6.7)	16 (8.4)	18 (15.3)	
How likely do you think it is that someone will die if they get breast cancer?						
Fatal disease	114 (24.2)		38 (25.5)	43 (22.5)	30 (25.4)	0.862
Probably	200 (42.5)		66 (44.3)	80 (41.9)	49 (41.5)	
Low probability	157 (33.3)		45 (30.2)	68 (35.6)	39 (33.1)	
What extent do you think that early detection of breast cancer will influence a person´s chance of surviving the breast cancer?						
A lot	398 (84.0)		125 (85.0)	163 (84.5)	100 (82.6)	0.858
Not much/nothing	76 (16.0)		22 (15.0)	30 (15.5)	21 (17.4)	
Do you think you can have breast cancer without having a lump or nodule?						
Yes	232 (50.9)		81 (57.4)	96 (51.6)	50 (42.7)	0.062
No	224 (49.1)		60 (42.6)	90 (48.4)	67 (57.3)	
If a woman finds a lump or nodule in her breast, would you say the chances of it being breast cancer are low, moderate or high?						
High	151 (32.1)		63 (42.6)	55 (28.8)	27 (22.7)	
Moderate	259 (55.0)		68 (45.9)	105 (55.0)	79 (66.4)	0.003
Low	61 (13.0)		17 (11.5)	31 (16.2)	13 (10.9)	
Knowledge						
Good	152 (32.8)		79 (53.7)	62 (32.8)	09 (7.6)	<0.001
Fair	175 (37.7)		54 (36.7)	86 (45.5)	32 (26.9)	
Poor	137 (29.5)		14 (9.5)	41 (21.7)	78 (65.5)	

*, Total may change due to missing values; **, Cutoff for high belief (>11 points); intermediate (from 7 to 11); low (< 7 points).

Frequencies of interviewees with high, intermediate, and low belief scores was 32.3%, 41.7%, and 26.0%, respectively. Belief scores categorized as high, intermediate, and low based on sociodemographic and epidemiological characteristics, health care, women’s experiences and understanding of breast cancer, and knowledge of the risk factors and disease signs/symptoms are presented in Table 3. High belief scores were statistically more frequent among women from 40 to 49 years (39.3%) and 50 to 59 years (32.7%), as compared with other age groups. High belief frequency was statistically higher among of women who had seen a gynecologist in the past 2 years had scores as compared with those who had not. Among women with high belief the frequency of believing that the likelihood of breast lump being breast cancer was statistically higher (42.6%) as compared to women with moderate (28.8%) and low (22.7%) beliefs.

## Discussion

Since 2008, the World Health Organization has recommended greater attention be paid to social determinants in public health research (CSDH, 2008). Culture is a social determinant of health that acts at the community and population levels. Culture includes beliefs, customs, social behaviors, attitudes, and traits of a particular social group (Licqurish et al., 2017). In the present study, the influence of culture on women’s beliefs about the risk factors for breast cancer was evident. 

Accordingly, 95% of women in this study reported that breast trauma increased the risk of developing breast cancer, and 82.6% of women believed that breast trauma could significantly increase the risk of breast cancer. Similarly, a hospital-based study conducted in Trinidad and Tobago, a country first inhabited by Amerindians with a slave cultural heritage influenced by Spanish, Indian, Chinese, and Portuguese cultures, found that approximately two-thirds (63.7%) of the respondents believed that breast trauma was a risk factor for breast cancer (Gosein et al., 2014). In both studies, most respondents believed that breast trauma was a risk factor for breast cancer, potentially due to the similar cultural heritage of these Latino countries, which were past colonies. However, the notion that breast injury leads women to seek specialized care, including screening tests, which often detect preexisting lesions, may contribute to the spread of the belief that breast trauma increases the risk of breast cancer (Ballesio et al., 2012). 

The hypothesis of the influence of culture on this specific belief is corroborated by a population-based study in 2,000 involving 996 British women, wherein only 28% believed that breast trauma or injury was a risk factor for the disease (Grunfeld et al., 2002). In Britain, where the cultural background is sustained by rational thinking, a low percentage of the female respondents thought that breast trauma increased the risk of breast cancer. A study conducted in Egypt with women recently diagnosed with breast cancer found that 37.78% of the interviewees believed that breast trauma was a risk factor for cancer (El-Shinawi et al., 2013). Despite the strong cultural heritage and the strong influence of religion in Egypt, a low percentage of women thought that breast trauma was a risk factor for cancer. This result might be because the study included women diagnosed with breast cancer who probably acquired more information about the disease. The different results of these studies indicate that the beliefs about the risk of breast cancer are strongly correlated with social and cultural factors, including the health history and experiences of these women.

Similarly, the majority of women in the present study(58.5%) believed that wearing underwire bra increased the risk of breast cancer. In Tanzania, a study found that a sample of 225 low-income women believed that putting money in her bra (82.2%) and regular use of a bra (18.2%) caused breast cancer (Morse et al., 2014). Similarly, a study from Ghana with 474 women aged 40 to 70 years reported that 20% thought that putting coins in their bra increased the risk of developing the disease (Opoku et al., 2012). A possible explanation for these findings is that similar to breast trauma, irritation of the breasts by an underwire or coin increases the probability of palpation of the breast, which may increase the probability of diagnosis of the disease (Ballesio et al., 2012). 

Although controversy exists regarding whether psychosocial factors are associated with cancer risk, many women believe that these factors affect breast cancer risk. In the present study, stress (68.9%) was the most cited psychosocial factor followed by depression, anger, or bitterness (62.5%) and personal disappointment (55.8%). Similarly, a study involving female Chinese immigrants aged **>** 40 years in the United States found that 82% of respondents believed that stress could increase the risk of developing breast cancer. In that same study, 55% of the evaluated women believed that having a positive mental attitude could reduce the risk of the disease (Lee-Lin et al., 2007). Although there is no strong evidence regarding the contribution of these factors to breast cancer development, some studies suggest that psychological factors, including bitterness and resentment, could explain how an emotional circumstance considered unfavorable triggers the onset of breast cancer (Dumalaon-Canaria et al., 2014; Kadhel et al., 2016). Dumalaon-Canaria (2014) conducted a systematic review and selected quantitative and qualitative studies that reported causal attributions or beliefs in breast cancer patients. Some women from the qualitative studies believed that breast cancer might occur after experiencing sad episodes, especially those involving bitterness. Among the nine quantitative studies included in this review, the prevalence of the belief that breast cancer was caused by characteristics associated with the personality of women (inability to cope with stressful situations, negative mental behavior, anxiety, depression, and not expressing feelings, among others) ranged from 2.6% to 35.0%, whereas the prevalence of the belief that stress was the leading cause of breast cancer was 68,9%. However, although some studies have attempted to associate breast cancer with psychosocial factors (Lillberg et al., 2002; Kadhel et al., 2016), there is no consensus to date on which of these factors and to what extent these factors may contribute to the development of breast cancer.

Sexual practices have also been identified as risk factors for breast cancer. Among those interviewed, 55.5% believed that a high number of sexual partners increased the risk of the disease. A possible explanation for this finding involves the dissemination by health education campaigns indicate that this practice is a risk factor for cervical cancer, potentially prompting women in this group to extrapolate this information to an increased risk of breast cancer(International Collaboration of Epidemiological Studies of Cervical Cancer, 2009). 

In the present study, women in the age group 50–59 years presented a higher average belief score compared with women in other age groups(p=0.005). Considering that the Brazilian Ministry of Health advocates that all women aged **>** 50 years undergo a mammography every 2 years as the primary strategy to decrease the incidence and mortality from breast cancer, it is of great importance to consider the beliefs about cancer in the planning of educational programs to increase the adherence of women in this age group to screening programs (Fugita and Gualda, 2006). The cultural beliefs and values of a population at the individual and social levels affect the health behavior of individuals (Leininger and McFarland, 2002).

The results of the present study indicate the existence of central beliefs related to the risk of breast cancer, and these beliefs may be affected by the strong cultural miscegenation in this Brazilian region. The fact that women with high belief scores believe that the probability of breast cancer is high if the woman finds a breast lump (B coefficient=0.79, p<0.001) and that it is possible to develop breast cancer without having a lump or nodule in the breast (B coefficient=1.05, p=0.007) suggests a mix of cultural beliefs and the scientific knowledge disseminated by the media and health services. This hypothesis is supported by the positive and significant association between knowledge scores and belief scores (B coefficient=0.335, p<0.001). 

The strong association between beliefs and scientific knowledge about the risk factors and signs and symptoms of breast cancer may be explained by the beliefs and experiences of Amerindians combined with the beliefs and scientific knowledge brought by Portuguese, Turks, and Lebanese immigrants since the beginning of colonization (Tocantins, 1979), which molded the beliefs about breast cancer among women from Rio Branco over the years. Although the scientific knowledge has been widely disseminated and generalized, these findings suggest that the beliefs inherited from older generations over the years are rooted in popular beliefs. Although no ethnographic study evaluated the effect of this cultural syncretism on the set of health beliefs of the population of Rio Branco to date, the possible relationship between current beliefs and the influence of knowledge/beliefs inherited from different cultures is supported by studies with other populations, including African Americans (Bailey, 1987). Bailey (1987) investigated the sociocultural factors that could affect the health care-seeking behavior in an African-American population using ethnographic data analyzed via the observation-participation method. The results indicated significant differences in health care behavior between the ethnic groups regarding health knowledge, attitudes toward health therapies, health-care seeking behavior, and the use of local health care services. 

Issues could arise from the limitations related to comparing our findings with the literature because there is a lack of studies that measure the relationship between scientific knowledge and beliefs about health and disease in the population. However, there are theoretical hypotheses that support and explain our findings. According to Herzlich (2005), the history of medicine indicates that both medical knowledge and lay knowledge have been reciprocally constructed and that no vertical relationship exists between “those who construct knowledge” and “those who receive and reinterpret knowledge.” 

In the epistemological debates about knowledge, it is common for contemporary epistemologists to consider beliefs as a necessary but not sufficient condition for knowledge (Chibeni, 2006). However, Hume (2000) considers that belief is not an essential condition for knowledge. This author considers that experience plays a direct epistemic role in constructing beliefs and an indirect role in constructing knowledge. In addition, experience serves only as a source of ideas. For other authors, there is an interrelationship between belief and knowledge with either primary or complementary function (Chisholm, 1966) that could justify the fact that the women from our study with greater knowledge about breast cancer also presented higher belief scores. Considering that epistemological issues on the process of formation of beliefs and knowledge are complex and that many theories from different thinkers seek to explain this relationship, a detailed investigation of these findings is necessary. Notwithstanding, changes in the health behavior of a population, including adherence to breast cancer screening, may be affected by whether health education programs disregard the set of beliefs acquired by this population.

One of the limitations of this study was the use of a scale that was not widely used in other studies, thus limiting the comparison with the findings from other populations. Notwithstanding, the scale used in this study was based on the previously validated Health Information National Trends Survey(HINTS) (National Cancer Institute, 2003). The strengths of this study include the fact it is the first to use a population-based quantitative approach to evaluate women’s beliefs about breast cancer in Brazil. Most of the studies on this subject in Brazil used qualitative methods and evaluated selected populations. Moreover, this study is the first to evaluate this topic in the western Amazon region, home to the largest indigenous population in Brazil despite the strong miscegenation. Finally, its large sample size is another strength, as it provided sufficient power to reliably estimate the belief scores and the frequency of the most common beliefs in the population of women aged **>** 40 years in Rio Branco, Acre.
